# Facile Non-Enzymatic Electrochemical Sensing for Glucose Based on Cu_2_O–BSA Nanoparticles Modified GCE

**DOI:** 10.3390/s19122824

**Published:** 2019-06-24

**Authors:** Zhikuang Dai, Ailing Yang, Xichang Bao, Renqiang Yang

**Affiliations:** 1 Department of Physics, College of Information Science and Engineering, Ocean University of China, Qingdao 266100, Shandong, China; dzkwymailbox@163.com; 2 Qingdao Institute of Bioenergy & Bioprocess Technology, Chinese Academy of Sciences, Qingdao 266100, Shandong, China; baoxc@qibebt.ac.cn

**Keywords:** Cu_2_O–BSA nanoparticles, non-enzymatic glucose sensing, cyclic voltammetry, chronoamperometry, anti-interference

## Abstract

Transition-metal nanomaterials are very important to non-enzymatic glucose sensing because of their excellent electrocatalytic ability, good selectivity, the fact that they are not easily interfered with by chloride ion (Cl^−^), and low cost. However, the linear detection range needs to be expanded. In this paper, Cu_2_O–bovine serum albumin (BSA) core-shell nanoparticles (NPs) were synthesized for the first time in air at room temperature by a facile and green route. The structure and morphology of Cu_2_O–BSA NPs were characterized. The as-prepared Cu_2_O–BSA NPs were used to modify the glassy carbon electrode (GCE) in a Nafion matrix. By using cyclic voltammetry (CV), the influence from scanning speed, concentration of NaOH, and load of Cu_2_O–BSA NPs for the modified electrodes was probed. Cu_2_O–BSA NPs showed direct electrocatalytic activity for the oxidation of glucose in 50 mM NaOH solution at 0.6 V. The chronoamperometry result showed this constructing sensor in the detection of glucose with a lowest detection limit of 0.4 μM, a linear detection range up to 10 mM, a high sensitivity of 1144.81 μAmM^−1^cm^−2^ and reliable anti-interference property to Cl^−^, uric acid (UA), ascorbic acid (AA), and acetaminophen (AP). Cu_2_O–BSA NPs are promising nanostructures for the fabrication of non-enzymatic glucose electrochemical sensing devices.

## 1. Introduction

Diabetes mellitus is a group of lifelong metabolic diseases characterized by hyperglycemia caused by multiple causes. According to the International Diabetes Federation (IDF) [1], in 2017, there is one diabetic patient for every 11 people aged 20–79, and 425 million people suffering from diabetes worldwide. It is estimated that the number of diabetic patients in this age group will rise to 629 million by 2045. At present, there is no cure for diabetes mellitus. People can only use blood sugar detection, diet therapy, exercise therapy, and drug treatment to control blood sugar level. To control blood sugar concentration, people must monitor blood sugar frequently, so blood sugar detection plays a vital role in the treatment of diabetes mellitus. This encourages researchers to explore the field of glucose biosensors.

Many methods have been used for glucose sensing, including electrochemical [2,3,4,5,6,7,8,9,10], chemiluminescence [11,12,13], colorimetry [2,14], Raman spectra [15,16], and fluorescence spectrometry [17,18,19,20,21]. In these methods, electrochemical glucose sensors are very important, because this method is simple and selective, along with having low detection limit, fast response time, good stability and low cost [2,3,4,5,6,7,8,9,10]. The geometric structure of an electrode surface is very important in an electrochemical glucose sensor. Nanomaterials have larger specific surface areas than that of bulk materials, so nanomaterials are often used to modify electrodes to provide larger surface areas, thus providing more adsorption sites and improving catalytic efficiency [4].

Enzyme-based glucose sensors are mainly used for blood glucose detection in the market, but the stability of enzyme is not good, which leads to the high price of this type of sensor [4,5,6,7,8,9]. As an alternative strategy, non-enzymatic glucose sensors have many advantages. They are almost free from the influence of oxygen, with good thermal and chemical stability, simple fabrication process, low cost, and good reproducibility, so are more conducive to miniaturization and large-scale production [4].

Noble metals of Au, Pt, Pd, and other precious metals have good conductivity, which gives them good performance in detecting glucose [22,23,24]. The chemical activity, conductivity, and surface roughness of Pt and Au electrodes are very good, but they lose their activity easily, due to the influence of Cl^−^ [22,23]. The cost of these noble electrodes is also expensive. Transition metals are low cost and can be used as electrocatalytic materials for glucose. There are many electrocatalysts reported for transition metals, including pure metals [6,25], bimetals [7,26,27,28], compounds [29,30,31,32], and composites [33,34,35,36,37,38,39,40,41,42,43,44] for non-enzymatic glucose sensors. The catalytic principle of non-enzymatic glucose sensors based on transition metals is by using the d-electron of d-orbital to form medium-strength bonds with substrates, so that the analyte can be easily adsorbed at any time, and its products can be easily desorbed [3]. 

The most common transition-metal materials are Ni [8] and Cu [9]. Ni and Cu have good selectivity and are not easily interfered by Cl^−^, but they must be effectively used in alkaline conditions. To be widely used, transition-metal materials such as copper and copper oxide are very good choices. The principle of electrocatalytic oxidation of Cu for glucose is similar to that of Ni. The catalytic effect lies in the redox reaction in redox pairs of Cu(II)/Cu(III) [45,46]. The electrode sensitivity of electrochemical glucose sensor based on Cu is also very high, but the linear range is not very wide. In addition, non-enzymatic glucose sensors based on copper material have excellent anti-interference, ultra-low detection limit, and low price. Justice Babu et al. [36] assumed 3D dendrite Cu-Co/reduced graphene oxide architectures on a disposable pencil graphite electrode as an electrochemical sensor for non-enzymatic glucose detection. Huang et al. [35] probed copper oxide (CuO) deposited oxygen-doped nitrogen incorporating nanodiamond (NOND)/Si pyramid (Pyr-Si) heterostructure for high-performance non-enzymatic glucose sensor. The growth time of CuO/NOND/Pyr-Si sensor is only 15 min, with a high sensitivity of 1993 μA mM^−1^ cm^−2^, a lower limit of detection of 0.1 μM, a linear range 5–700 μM, a rapid recovery (<2 s) and longer stability of 28 d (∼96%). The sensor exhibits good selectivity of glucose among other analytes such as Cl^−^, AC, UA, and so on. Gou et al. [47] synthesized ultrathin CuO nanorods by simply mixing Cu^2+^/OH^−^/ethanol with water at 75 °C towards non-enzymatic glucose sensing. Such 1D CuO nanorods acting as a promising electrode material show substantially good performance. A high sensitivity of 1834 μA mM^−1^ cm^−2^, a fast response time of 2 s, a relatively wide linear dynamic range from 100 μM to 4.0 mM, a low detection limit of 220 nM and good anti-interference ability were demonstrated. Sahoo et al. [48] grew faceted and non-faceted CuO nanoribbons via hydrothermal and microwave heating processes. The glucose sensitivity of a faceted CuO electrode is superior to that of a non-faceted CuO one. The electrochemical glucose detection of the faceted CuO electrode shows a minimum concentration of 58 μM and a specific current sensing of 412 μA mM^−1^cm^−2^, whereas the non-faceted CuO electrode shows low sensitivities of 71 μM and 356 μA mM^−1^cm^−2^. Cheng et al. [33] prepared a 3D network and 2D paper of reduced graphene oxide/Cu_2_O composite for electrochemical sensing of H_2_O_2_ in human serum. The present study indicates that 2D and 3D graphene–Cu_2_O composites have promising applications in the fabrication of non-enzymatic electrochemical sensing devices. Dong et al. [34] synthesized a unique binder-free electrode with the nanoarchitecture of 3D Cu foam/mesoporous Cu_2_O nanothorn arrays through a facile two-step anodization/electro-oxidation procedure. This enzyme-free electrochemical electrode exhibits ultra-high sensitivity maximum of 97.9 mA mM^−1^cm^−2^, an ultra-low detection limit (5 nM) and excellent selectivity and stability. The Cu foam-supported Cu_2_O electrode can meet the glucose detection for human serum over a wide concentration range (1 μM to 11.4 mM). Our group fabricated hollow Cu_2_O–PVP NPs by stepwise controlling the addition of the reducer [49] and constructed a non-enzymatic glucose sensor based on the hollow Cu_2_O nanosphere in Nafion matrix on a glassy carbon electrode (GCE) [50]. Results showed that the hollow Cu_2_O nanospheres have a remarkable electrocatalytic activity for the oxidation of glucose without loading any enzymes. The sensor exhibited high sensitivity, low detection limit, and strong anti-interference property, but the linear detection range is narrow (1.25–37.5 μM).

It is known that the catalytic performance of the nanomaterials depends on their surface areas, surface atomic structures, and morphologies. By controlling the shapes, sizes, and coating agents of the nanomaterials, catalysts with large surface areas and abundant catalytic active sites can be obtained, thereby improving catalytic performance. As a model globular protein, bovine serum albumin (BSA) is widely used in biochemical studies [51]. BSA is composed of 583 amino acid residues, several sulfur, oxygen, and nitrogen atoms [51]. In recent years, BSA were generally used to stabilize the inorganic NPs [51,52,53,54,55,56]. BSA-stabilized Au and Ag NPs have been widely explored for biomedical [54,55,56,57,58,59] and sensing applications [60]. The fabrication of metal–BSA NPs is a green reaction process. The products demonstrated excellent conductivity, biocompatibility, and multifunctionality, and were environmentally friendly and highly stable in both aqueous and solid form. Hu et al. [61] prepared Pt–BSA nanocomposite as an enzyme-labeled glucose-sensing interface. 

In this paper, Cu_2_O–BSA NPs with size of 180–220 nm was prepared via a green route and characterized by TEM and XRD. Taking advantage of as-prepared Cu_2_O–BSA NPs as catalyst and microscopic network structure of Nafion to stabilize the modified electrode, a novel non-enzymatic glucose sensor was constructed. Results showed that the Cu_2_O–BSA NPs exhibit a remarkable electrocatalytic activity and electrochemical sensing matrix for the oxidation of glucose, free from loading any enzymes. The modified electrode indicated wide linear detection range of up to 10 mM, high sensitivity, low detection limit, fast response time, and strong anti-interference property to Cl^−^, AA, UA, and AP. Our results indicated that the Cu_2_O–BSA NPs is an excellent material for non-enzyme electrochemical biosensors.

## 2. Materials and Methods

### 2.1. Reagents and Characterization

Copper nitrate trihydrate (Cu(NO_3_)_2_⋅3H_2_O), sodium hydroxide (NaOH), sodium chloride (NaCl), potassium ferricyanide, potassium ferrocyanide, glucose, ethanol, AA, UA, and AP are analytical pure and were bought from Sinopharm Chemical Reagent Co., Ltd. BSA was purchased from Aladdin with purity of 96%. Nafion 117 solution is analytical purity, produced by Sigma-Aldrich Company, USA. All of reagents were used without further purification.

The crystalline structure of Cu_2_O–BSA NPs was detected by an X-ray diffractometer (Bruker D8 ADVANCE) with Cu-Kα radiation (λ = 1.5406 Å). A type of H-7650 TEM (Japan) was used to observe the size and morphology of the Cu_2_O–BSA NPs. A CHI 690 electrochemical workstation (Chenhua, Shanghai, China) was assumed to conduct the electrochemical experiments including CV and amperometry. A conventional three-electrode electrochemical system, which consists of a working electrode, a Pt foil auxiliary electrode, and Ag/AgCl reference electrode, was used for all electrochemical experiments.

### 2.2. Synthesis of the Cu_2_O–BSA NPs

70 mg BSA was dissolved in 26.4 mL deionized water with magnetic stirring for 10 min. 1.4 mL 0.2 M Cu(NO_3_)_2_ solution was added to BSA solution and stirred for 1 min, then 2.2 mL 1 M NaOH solution was added to the above solution and stirred for 5 min. 5 mL 0.1 M AA was added dropwise into the solution for reduction of Cu^2+^. After stirring for 10 min, the solution became orange colloid and the color was stable, which indicated Cu_2_O–BSA NPs were generated. The colloid was aged for half an hour then centrifuged three times at 8000 rpm for 10 min. The final Cu_2_O–BSA NPs were freeze dried in a vacuum freeze dryer and sealed in a small brown glass bottle, then preserved in a refrigerator at 4 °C for later use.

### 2.3. Preparation Modified Electrode of Nafion/Cu_2_O–BSA/GCE

Typically, 5 mg Cu_2_O–BSA NPs was added into 1 mL ethanol and sonicated for 15 min. 10 µL dispersion liquid of Cu_2_O–BSA NPs was dropped on the surface of bare treated GCE (diameter, 3 mm). After drying in air at room temperature, Cu_2_O–BSA NPs were evenly coated on the surface of GCE. Then 5 µL 0.1 wt. % Nafion/ethanol solution was dropped on the coating of Cu_2_O–BSA NPs. After natural drying, the modified electrode was sealed and stored for later use. The as-prepared electrode was named Nafion/Cu_2_O–BSA/GCE. For comparison, a Nafion/GCE without modified Cu_2_O–BSA NPs was also prepared by the same process.

## 3. Results and Discussion

### 3.1. Structure and Morphology of Cu_2_O–BSA NPs

**Figure 1:**
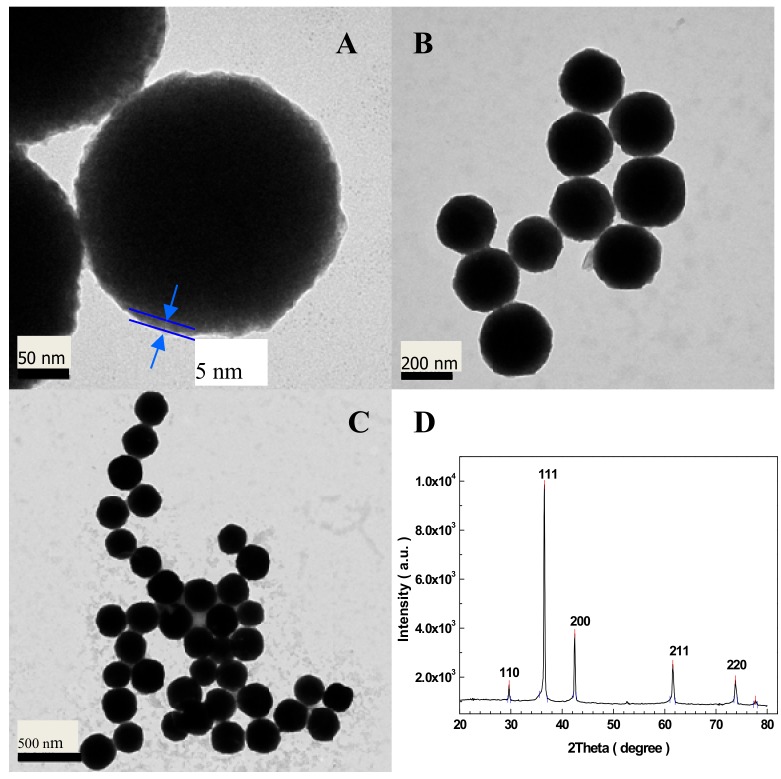
TEM images (**A**–**C**) and XRD pattern (**D**) of Cu_2_O–BSA NPs. The red lines in D are the standard peaks (PDF No. 65-3288) of Cu_2_O.

Figure 1A–C indicate the TEM images of Cu_2_O–BSA NPs. One can see that the NPs are spherical with a size of 180–220 nm. There is a thin layer of about 5 nm of BSA on the surface of Cu_2_O NP (see Figure 1A). So, the structure of the Cu_2_O–BSA NP is a core-shell structure. The NPs have good monodispersity. Figure 1D shows the powder XRD pattern of the Cu_2_O–BSA NPs. The five peaks localized at 29.64°, 36.54°, 42.50°, 61.59°, and 73.78° coincide well with the (110), (111), (200), (211), and (220) planes of the standard data (PDF No. 65-3288) for the cubic phase of Cu_2_O. According to Deby–Scherer equation [62], the spacing of crystal planes was calculated as follows: D_110_ = 2.6976 nm, D_111_ = 3.3633 nm, D_200_ = 3.9559 nm, D_211_ = 5.0383 nm, and D_220_ = 6.0699 nm.

### 3.2. Electrochemical Characterization of the Sensor

**Figure 2:**
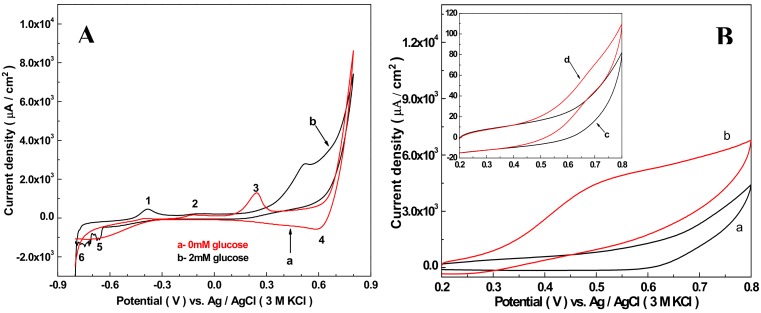
(**A**) Redox peaks of the present Nafion/Cu_2_O–BSA/GCE in the 50 mM NaOH solution with 0 (a) and 2 mM (b) of glucose at the scan rate of 5 mV/s, (**B**) Nafion/Cu_2_O–BSA/GCE and Nafion/GCE (inset) in the absence (a and c) and presence (b and d) of 5.0 mM glucose at the same pH with scanning speed of 50 mV/s.

It is reported that the Cu_2_O/MWCNTs modified electrode [63] works well in an alkaline environment for electrochemical oxidation glucose. Here 50 mM NaOH solution was used to probe electrocatalytic activities of the Nafion/Cu_2_O–BSA/GCE electrode towards the oxidation of glucose. The result is shown in Figure 2A, in which curve a and b represent 0 and 2 mM glucose in the solution, respectively. The scan rate is 5 mV/s. In the absence of glucose (curve a), the valence changes of copper ion is represented by the five peaks that can be determined according to [64], with Peak 1 near to −0.375 V representing the oxidation of Cu(0) to Cu(I). Peak 1 is very low because the material modified GCE is Cu_2_O–BSA NPs, the content of copper (0) is zero. Peak 2 at −0.110 V refers to the oxidation process of Cu(I) to Cu(II). This peak is also low, which indicates that the transformation of Cu(I) to Cu(II) is slow. The formation of peak 3 at 0.25 V is related to the adsorption of OH^−^ on the electrode and the formation of soluble substances from Cu_2_O-based solids. However, the potential of peak 3 is much higher than that of reference [65] and reference [66]. We speculate the formation of Peak 3 may be related to the negative charge of BSA, which leads to the voltage of the electrode-adsorbed OH^−^ shifting a lot towards the right. According to reference [54], in high-concentration sodium hydroxide solution, Cu(III) begins to form at 0.6 V. Peak 4 at 0.6 V attributes to the reduction process from Cu(III) to Cu(II). Peak 5 indicates the reduction from Cu(II) to Cu(I). In the whole cycle, no Cu(I) is converted to Cu(0), which means that no Cu(0) was formed during the cycle. When 2 mM glucose was added into solution, Peak 1 became obvious, which indicates Cu (0) was formed during the cycling process. Peak 2 is very low and almost the same as the absence of 2 mM glucose, which indicates that a small quantity of Cu(0) was generated before this stage, and most Cu(0) has been oxidized to Cu(I) during the period of peak 1 to peak 2. The disappearance of peak 3 may be the formation of complex (Cu(I)-glucose) on the surface of the electrode by the adsorption of glucose, which hinders the formation of soluble substances between Cu_2_O and OH^−^. A new oxidation peak at about 0.52 V is due to the desorption of glucose from the Cu(I)–glucose complex and the simultaneous oxidation of Cu(I) to Cu(II). The reason of the disappearance of peak 4 is the consumption of Cu(III) by electric oxidation of glucose. Peaks 5 and 6 are the reduction processes of Cu(II) to Cu(I) and Cu(I) to Cu(0), respectively.

The electrocatalytic effect of the Nafion/Cu_2_O–BSA/GCE was further compared with the Nafion/GCE for oxidation of glucose, as shown in Figure 2B. For the Nafion/Cu_2_O–BSA modified GCE, curve a and b show the CV results of absence and presence 5 mL glucose. The peak current density ratio of curve b and curve a is 1.54, which indicates the current density increase when glucose was catalyzed on the surface of Nafion/Cu_2_O–BSA/GCE. For the only Nafion-modified GCE, curve c and d in inset indicate the results of CV without and with 5 mL glucose. The peak current density ratios of curve d and curve c is 1.2, which shows the current density of only Nafion-modified GCE also increases when glucose was catalyzed on the surface of Nafion/ GCE. However, the extent of increase of Nafion/GCE is far less than that of the Nafion/Cu_2_O–BSA/ GCE because the current density of the Nafion/Cu_2_O–BSA/GCE is 60 times of the Nafion/GCE. The above results indicate Cu_2_O–BSA NPs greatly increase the catalytic ability of the GCE for glucose, which is ascribed to high catalytic active sites for the glucose oxidation offered by the Cu_2_O NPs and good conductivity of BSA.

**Figure 3:**
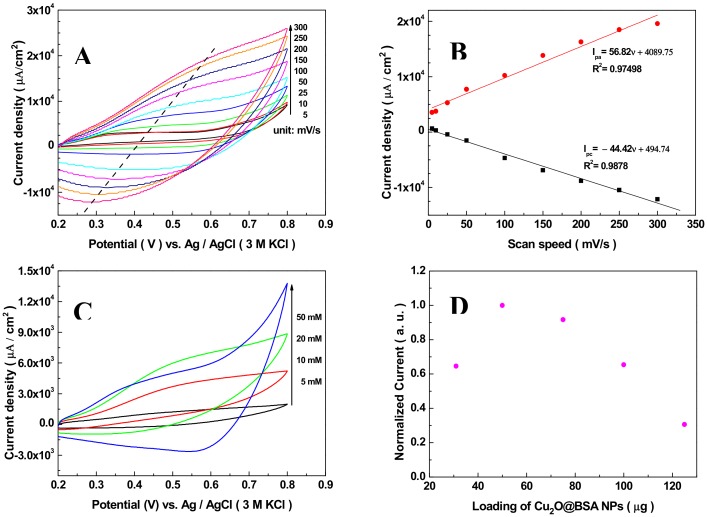
(**A**) CVs of the Nafion/Cu_2_O–BSA/GCE in 50 mM NaOH solution with 2 mM of glucose at different scan rates, (**B**) Linear dependence of oxidation and reduction peak current densities in (A) with the scan rates, (**C**) CVs at different concentrations of NaOH, (**D**) Current density changing with the loads of Cu_2_O–BSA NPs modified on the GCE. 2 mM of glucose and 50 mV/s scan rate in (C) and (D).

It is known that the scanning rate, concentration of NaOH in electrolyte, and load of Cu_2_O NPs on the GCE have effects on the current density of CVs. To obtain an optimal result, the above factors were probed, respectively. Figure 3A displayed the CVs of the Nafion/Cu_2_O–BSA/GCE for glucose oxidation recorded in 50 mM NaOH solution with the scanning speed in the range of 5–300 mV/s. According to Figure 3A, the peak current densities of oxidation (red line) and reduction (black line) and their change with the scanning speeds are shown in Figure 3B. The oxidative and reduction peak current densities are proportional to the scan rates in the range of 5–300 mV/s, with correlation coefficients (R^2^) of 0.9878 and 0.9750 (see Figure 3B), indicating that the electrocatalytic oxidation of glucose by the Nafion/Cu_2_O–BSA/GCE under alkaline conditions is a surface-controlled process similar to the previous report [67]. 50 mV/s was selected as the scanning speed in later experiments. Figure 3C shows the CV results when the concentrations of NaOH are 5, 10, 20, and 50 mM with same concentration of glucose (2 mM) and at the same scan rate (50 mV/s). The current densities of CVs increase with the concentrations of NaOH. This is because the conductivity of electrolyte increases at higher pH and the mutarotation of glucose becomes easy with the increase of pH [63], which speeds up the electrocatalytic oxidation of glucose, and more Cu(III) was produced. 50 mM NaOH was selected as the fixed concentration for later experiments.

In our experiments, 30–125 μg load of Cu_2_O–BSA NPs was used to modify the GCEs. With the increase of the NPs loads, the current densities increase first then decrease. When the load of Cu_2_O NPs is 50 μg, the maximum current density was obtained. The reason for the above results is that more absorption sites and catalyzing sites are supplied with the increase of Cu_2_O NPs load, but excessive load of NPs on the GCE leads to the increase of electrode resistance and the decrease of current. The optimal load of Cu_2_O NPs of 50 μg was assumed in later experiments.

### 3.3. Amperometric Response of the Sensor Towards Glucose

**Figure 4:**
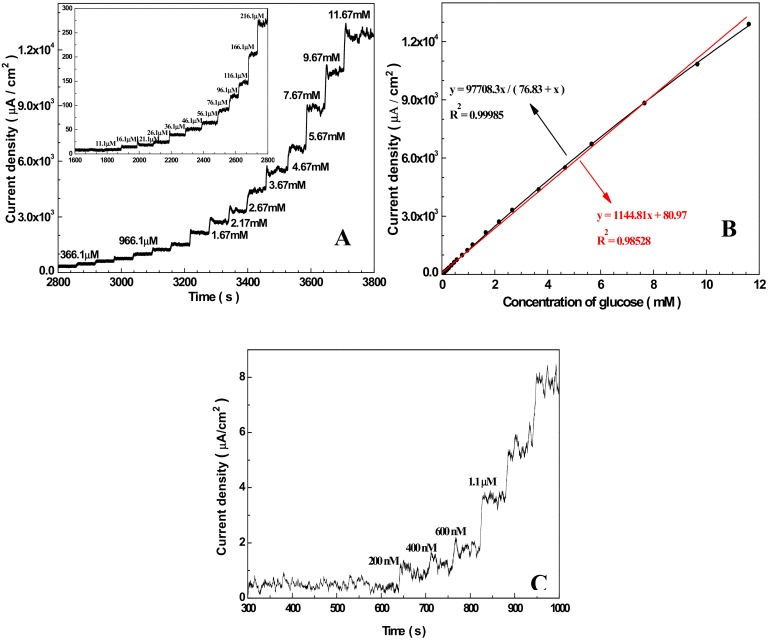
Chroamperometric response (**A**), calibration curve (**B**) and detection limit (**C**) of Nafion/Cu_2_O–BSA/GCE for successive addition of various concentrations of glucose to 50 mM NaOH solution at 0.6 V.

Figure 4A shows a chronoamperometric diagram of the Nafion/Cu_2_O–BSA/GCE responding to successive addition of glucose at a work potential of 0.6 V (vs. Ag/AgCl). The concentrations of glucose in the electrolyte are in the range or 200 nM−11.67 mM. For clarity, the chronoamperometric curve when the concentrations of glucose are in the range of 11.1 μM−216.1 μM is shown in the inset. Figure 4B presents the calibration curve (black curve) for the Nafion/Cu_2_O–BSA/GCE based on the result of Figure 4A. The red line is the Langmuir isothermal fitting curve [68]. One can see the linear range is up to 10 mM and the sensitivity is 1144.81 µAcm^−2^mM^−1^. The corresponding regression equation is I (μA/cm^2^) = 1144.81C (mM) + 80.98 (μA/cm^2^) (R^2^ = 0.98528). When the concentration of glucose is very low, the current of the electrode is also very low, and the noise is very high. Therefore, a separate study was carried out. Figure 4C displays an enlarged chronoamperometric curve of the Nafion/Cu_2_O–BSA/GCE when the concentration of glucose is as low as 200 nM. One can see from Figure 4C that the lowest detection limit is 400 nM (S/N = 3), and the response time is about 7 s. Therefore, the Nafion/Cu_2_O–BSA/GCE has a wide linear detection range, a high sensitivity, a low detection limit, and a quick response time to glucose.

In comparison to the performance of Cu_2_O-modified GCE in recent years (Table 1), the Cu_2_O–B SA NPs modified GCE have high sensitivity and wide linear range. The higher sensitivity is attributed to high electrocatalytic activity of Cu_2_O–BSA NPs with large surface area, BSA has good adsorption ability to glucose and good conductivity to electrons [58,59,61]. In comparison with our previous hollow Cu_2_O–PVP/Nafion-modified GCE [50], the linear detection range of Cu_2_O–BSA increases by 267 times.

### 3.4. Specificity, Reproducibility, and Stability of the Nafion/Cu_2_O–BSA/GCE

**Figure 5:**
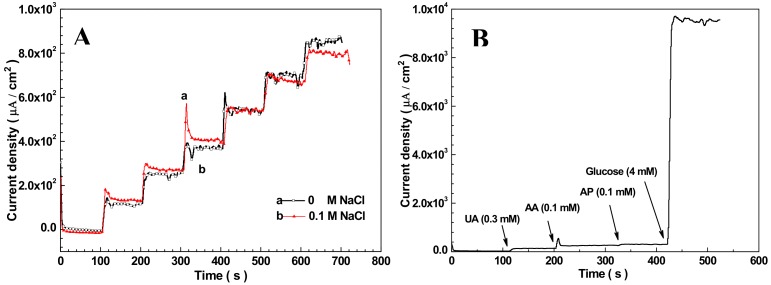
(**A**) Chroamperometric response of Nafion/Cu_2_O–BSA/GCE to glucose in 0 M (a) and 0.1 M (b) chloride ions; (**B**) Interference test of Nafion/Cu_2_O–BSA/GCE to 0.3 mM UA, 0.1 mM AA and 0.1 mM AP in 50 m NaOH at 0.6 V.

Cl^−^, UA, AA, and AP are common interference contents in human blood for EC glucose sensing. Their normal physiological concentrations are 0.1, 0.3, 0.1, and 0.1 mM, respectively. To detect the toxicity of Cl^−^ to Nafion/Cu_2_O–BSA/GCE, the chronoamperometric responding to the glucose was carried out when concentrations of Cl^−^ are 0 M and 0.1 M (up to 1000 times concentration in normal human blood) in the electrolyte. The potential is 0.6 V (vs. Ag/AgCl) and the NaOH concentration is 50 mM. The results are shown in Figure 5A. One can see that the two chronoamperometric curves responding to the glucose with and without 0. 1 M Cl^−^ have little difference, which indicates that Cl^−^ has little interference with the Nafion/Cu_2_O–BSA/GCE. Unlike Au and Pt electrodes [22,23], Cl^−^ has no toxicity to Nafion/Cu_2_O–BSA/GCE. To explore the interference from UA, AA, and AP on Nafion/Cu_2_O–BSA/GCE, 0.3 mM UA, 0.1 mM AA, 0.1 mM AP and 4 mM glucose (normal physiological level) were successive added to 50 mM NaOH solution for chronoamperometric detection. The results are shown in Figure 5B. The current response of Nafion/Cu_2_O–BSA/GCE to 4 mM glucose is 76, 75, and 180 times of the responses of 0.3 mM UA, 0.1 mM AA, and 0.1 mM AP, respectively. Therefore, UA, AA, and AP have quite limited influence on the detection of glucose. The Nafion/Cu_2_O–BSA/GCE has good specificity to glucose sensing.

**Figure 6:**
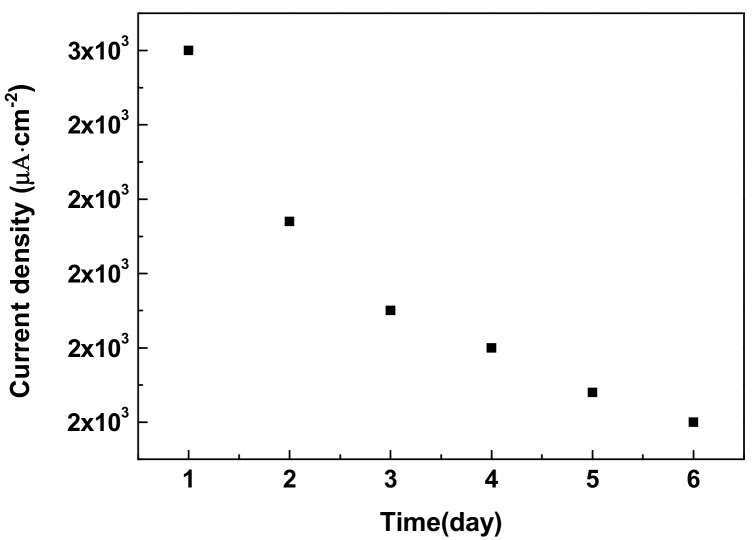
Amperometric response of Nafion/Cu_2_O–BSA/GCE to glucose over time.

The storage stability of Nafion/Cu_2_O–BSA/GCE was conducted over six days of consecutive testing, with 2 mM glucose in 50 mM NaOH solution for 6 min, and the voltage fixed at 0.6 V. After testing, the electrode was dipped in pure water for 3 min, three times, then taken out and dried in air. The electrode was stored at room temperature when not in use. The results are shown in Figure 6. For the same Nafion/Cu_2_O–BSA/GCE, the current response decreased over time. On the sixth day, the current density fell to 80% of that of the first day. In our previous experiment [50], Cu_2_O–PVP/Nafion-modified GCE had good stability for five weeks. We guess this result may be relative to the coating agent of BSA. BSA is a type of protein, which is easier influenced by environment than PVP. In addition, a group of six Nafion/Cu_2_O–BSA/GCEs were prepared by the same method and used to do the chronoamperometry experiments, a relative standard deviation (RSD) of 6.9% was obtained, which indicates good fabrication reproducibility of these sensing electrodes. The Cu_2_O–BSA NPs we prepared make good material for the fabrication of sensitive, specific, and rapid amperometric sensors for the non-enzymatic detection of glucose.

## 4. Conclusions

In this paper, Cu_2_O–BSA NPs were synthesized in air at room temperature by Cu(NO_3_)_2_⋅3H_2_O as a precursor, BSA as a coating agent, and AA as a reductant. The sphere NPs with size of 180–220 nm have good monodispersity. The as-prepared Cu_2_O–BSA NPs were used to modify the GCE. By using CV and chronoamperometry, Cu_2_O–BSA NPs showed direct electrocatalytic activity for the oxidation of glucose in 50 mM NaOH solution at 0.6 V. The Nafion/Cu_2_O–BSA NPs film provides rich active sites for the sensing of glucose. This constructed sensing electrode was used in the detection of glucose with the lowest detection limit of 0.4 μM, a linear detection range up to 10 mM, a high sensitivity of 1144.81 μAmM^−1^cm^−2^ and a quick response time of 7 s. The common interfering contents of Cl^−^, UA, AA, and AP existing in the physiological environment have almost no interference in the oxidation of glucose for the Nafion/Cu_2_O–BSA/GCE. For the green synthesis of Cu_2_O–BSA NPs and facile modification of GCE, there was excellent performance of the Nafion/Cu_2_O–BSA/GCE for glucose, and Cu_2_O–BSA NPs will become promising nanostructures for the fabrication of non-enzymatic glucose electrochemical sensing devices.

## Figures and Tables

**Table 1:** Comparison of sensing behavior of Nafion/Cu_2_O–BSA/GCE with other reported Cu_2_O or CuO-based non-enzymatic glucose sensors.

Electrode Material	Sensitivity (μA cm^−2^ mM^−1^)	Linear Range (mM)	Detection Limit (μM)	Year	Reference
Nafion/Cu_2_O–BSA/GC	1144.81	up to 10	0.4	2019	This Work
Cu_2_O NPs	2310 ± 30	0.00048–1.813	0.14 ± 0.01	2017	[69]
Cu_2_O/graphene/GCE	1330.05	0.01–3.0	0.36	2016	[70]
Cu_2_O NPs	507	0.1–2.5	26	2016	[71]
Cu_2_O/Nafion/GCE	2038.2	0.00125–0.0375	0.41	2015	[50]
Cu_2_O-CLGNs/GCE	1215.7	0.1–5	1.83	2015	[72]
CuO/Cu_2_O NFs	830	0.5–10	Not given	2014	[67]
Cu_2_O/Cu	728.67	0.53–7.53	Not given	2012	[73]
	3029.33	0.01–0.53			
Cu_2_O/Carbon Vulcan XC-72	629	up to 6	2.4	2011	[74]

## References

[1] Cho N.H., Shaw J.E., Karuranga S., Huang Y., da Rocha Fernandes J.D., Ohlrogge A.W., Malanda B. (2018). IDF Diabetes atlas: Global estimates of diabetes prevalence for 2017 and projections for 2045. Diabetes Res. Clin. Pract..

[2] Rahman M.M., Ahammad A.J.S., Jin J.H., Ahn S.J., Lee J.J. (2010). A comprehensive review of glucose biosensors based on nanostructured metal-oxides. Sensors.

[3] Gebhardt U., Luft G., Richter G.J., von Sturm F. (1978). Development of an implantable electrocatalytic glucose sensor. Bioelectrochem. Bioenerg..

[4] Park S., Boo H., Chung T.D. (2006). Electrochemical non-enzymatic glucose sensors. Anal. Chim. Acta.

[5] Toghill K.E., Compton R.G. (2010). Electrochemical non-enzymatic glucose wensors: A perspective and an evaluation. Int. J. Electrochem. Sci..

[6] Wang G.F., He X.P., Wang L.L., Gu A.X., Huang Y., Fang B., Geng B.Y., Zhang X.J. (2013). Non-enzymatic electrochemical sensing of glucose. Microchim. Acta.

[7] Si P., Huang Y.J., Wang T.H., Ma J.M. (2013). Nanomaterials for electrochemical non-enzymatic glucose biosensors. RSC Adv..

[8] Chen C., Xie Q.J., Yang D.W., Xiao H.L., Fu Y.C., Tan Y.M., Yao S.Z. (2013). Recent advances in electrochemical glucose biosensors: A review. RSC Adv..

[9] Tian K., Prestgard M., Tiwari A. (2014). A review of recent advances in nonenzymatic glucose sensors. Mater. Sci. Eng. C.

[10] Tee S.Y., Teng C.P., Ye E. (2017). Metal nanostructures for non-enzymatic glucose sensing. Mater. Sci. Eng. C.

[11] Zhu N.F., Gu L.T., Wang J., Li X.S., Liang G.X., Zhou J.H., Zhang Z. (2019). Novel and sensitive chemiluminescence sensors based on 2D-MOF nanosheets for one-step detection of glucose in human urine. J. Phys. Chem. C.

[12] Li H.J., Liu C.L., Wang D., Zhang C.S. (2017). Chemiluminescence cloth-based glucose test sensors (CCGTSs): A new class of chemiluminescence glucose sensors. Biosens. Bioelectron..

[13] Hao M.J., Liu N., Ma Z.F. (2013). A new luminol chemiluminescence sensor for glucose based on pH-dependent graphene oxide. Analyst.

[14] Qi L.B., Hu Q.Z., Kang Q., Yu L. (2018). Fabrication of liquid-crystal-based optical sensing platform for detection of hydrogen peroxide and blood glucose. Anal. Chem..

[15] Pandey R., Paidi S.K., Valdez T.A., Zhang C., Spegazzini N., Dasari R.R., Barman I. (2017). Noninvasive monitoring of blood glucose with Raman spectroscopy. Acc. Chem. Res..

[16] Yang D., Afroosheh S., Lee J.O., Cho H., Kumar S., Siddique R.H., Narasimhan V., Yoon Y.Z., Zayak A.T., Choo H. (2018). Glucose sensing using surface-enhanced Raman-mode constraining. Anal. Chem..

[17] Locke A.K., Means A.K., Dong P., Nichols T.J., Coté G.L., Grunlan M.A. (2018). A Layer-by-Layer Approach to Retain a Fluorescent Glucose Sensing Assay within the Cavity of a Hydrogel Membrane. ACS Appl. Bio Mater..

[18] Wang D., Pan K., Qu Y., Wang G.F., Yang X.F., Wang D.S. (2018). BaWO_4_:Ln^3+^ nanocrystals: Controllable synthesis, theoretical investigation on the substitution site, and bright upconversion luminescence as a sensor for glucose detection. ACS Appl. Nano Mater..

[19] Li J.M., Wu H.L., Santana I., Fahlgren M., Giraldo J.P. (2018). Standoff optical glucose sensing in photosynthetic organisms by a quantum dot fluorescent probe. ACS Appl. Mater. Interf..

[20] Pan Z.B., Wang Y.C., Chakkaradhari G., Zhu J.F., He R.Y., Liu Y.C., Hsu C.H., Koshevoy I.O., Chou P.T., Pan S.W. (2018). A silver metal complex as a luminescent probe for enzymatic sensing of glucose in blood plasma and urine. Dalton Trans..

[21] Zhang W.C., Li X., Xu X.C., He Y.F., Qiu F.X., Pan J.M., Niu X.H. (2019). Pd nanoparticle-decorated graphitic C_3_N_4_ nanosheets with bifunctional peroxidase mimicking and ON–OFF fluorescence enable naked-eye and fluorescent dual-readout sensing of glucose. J. Mater. Chem. B.

[22] Guo M.Q., Hong H.S., Tang X.N., Fang H.D., Xu X.H. (2012). Ultrasonic electrodeposition of platinum nanoflowers and their application in nonenzymatic glucose sensors. Electrochim. Acta.

[23] Sharma S., Gupta N., Srivastava S. (2012). Modulating electron transfer properties of gold nanoparticles for efficient biosensing. Biosens. Bioelectron..

[24] Meng L., Jin J., Yang G.X., Lu T.H., Zhang H., Cai C.X. (2009). Nonenzymatic electrochemical detection of glucose based on palladium-single-walled carbon nanotube hybrid nanostructures. Anal. Chem..

[25] Tian H.F., Jia M.Z., Zhang M.X., Hu J.B. (2013). Nonenzymatic glucose sensor based on nickel ionimplanted-modified indium tin oxide electrode. Electrochim. Acta.

[26] Noha H.B., Lee K.S., Chandra P., Won M.S., Shim Y.B. (2012). Application of a Cu-Co alloy dendrite on glucose and hydrogen peroxide sensors. Electrochimi. Acta.

[27] Li X.L., Yao J.Y., Liu F.L., He H.C., Zhou M., Mao N., Xiao P., Zhang Y.H. (2013). Nickel/copper nanoparticles modified TiO_2_ nanotubes for non-enzymatic glucose biosensors. Sens. Actuators B Chem..

[28] Tee S.Y., Ye E.Y., Pan P.H., Lee C.J., Hui H.K., Zhang S.Y., Koh L.D., Dong Z.L., Han M.Y. (2015). Fabrication of bimetallic Cu/Au nanotubes and their sensitive, selective, reproducible and reusable electrochemical sensing of glucose. Nanoscale.

[29] Babu K.J., Kumar T.R., Yoo D.J., Phang S.M., Kumar G.G. (2018). Electrodeposited nickel cobalt sulfide flowerlike architectures on disposable cellulose filter paper for enzyme-free glucose sensor applications. ACS Sustain. Chem. Eng..

[30] Sinha L., Pakhira S., Bhojane P., Mali S., Hong C.K., Shirage P.M. (2018). Hybridization of Co_3_O_4_ and α-MnO_2_ nanostructures for high performance nonenzymatic glucose sensing. ACS Sustain. Chem. Eng..

[31] Huang M., Luo X., He D., Jiang P. (2017). Hierarchical Co(OH)_2_ nanotube arrays grown on carbon cloth for use in non-enzymatic glucose sensing. Anal. Methods.

[32] Tomanin P.P., Cherepanov P.V., Besford Q.A., Christofferson A.J., Amodio A., McConville C.F., Yarovsky I., Caruso F., Cavalieri F. (2018). Cobalt phosphate nanostructures for non-enzymatic glucose sensing at physiological pH. ACS Appl. Mater. Interf..

[33] Cheng C.F., Zhang C.M., Gao X.H., Zhuang Z.H., Du C., Chen W. (2018). 3D network and 2D paper of reduced graphene oxide/Cu_2_O composite for electrochemical sensing of hydrogen peroxide. Anal. Chem..

[34] Dong C.Q., Zhong H., Kou T.Y., Frenzel J., Eggeler G., Zhang Z.H. (2015). Three-dimensional Cu foam-supported single crystalline mesoporous Cu_2_O nanothorn arrays for ultra-highly sensitive and efficient nonenzymatic detection of glucose. ACS Appl. Mater. Interf..

[35] Huang B.R., Wang M.J., Kathiravan D., Kurniawan A., Zhang H.H., Yang W.L. (2018). Interfacial effect of oxygen-doped nanodiamond on CuO and micropyramidal silicon heterostructures for efficient nonenzymatic. ACS Appl. Bio. Mater..

[36] Babu K.J., Sheet S., Lee Y.S., Kumar G.G. (2018). Three-dimensional dendrite Cu−Co/reduced graphene oxide architectures on a disposable pencil graphite electrode as an electrochemical sensor for nonenzymatic glucose detection. ACS Sustain. Chem. Eng..

[37] Zhu W.X., Wang J., Zhang W.T., Hu N., Wang J., Huang L.J., Wang R., Suo Y.R., Wang J.L. (2018). Monolithic copper selenide submicron particulate film/copper foam anode catalyst for ultrasensitive electrochemical glucose sensing in human blood serum. J. Mater. Chem. B.

[38] Chen X.R., Liu D., Cao G.J., Tang Y., Wu C. (2019). In Situ Synthesis of a sandwich-like graphene@ZIF-67 heterostructure for highly sensitive nonenzymatic glucose sensing in human serums. ACS Appl. Mater. Interf..

[39] Zhang Y.X., Xia J.F., Zhang F.F., Wang Z.H. (2018). MOF-derived porous Ni2P/graphene composites with enhanced electrochemical properties for sensitive nonenzymatic glucose sensing. ACS Appl. Mater. Interf..

[40] Deepalakshmi T., Tran D.T., Kim N.H., Chong K.T., Lee J.H. (2018). Nitrogen-doped graphene-encapsulated nickel cobalt nitride as a highly sensitive and selective electrode for glucose and hydrogen peroxide sensing applications. ACS Appl. Mater. Interf..

[41] Meng A., Sheng L.Y., Zhao K., Li Z.J. (2017). A controllable honeycomb-like amorphous cobalt sulfide architecture directly grown on the reduced graphene oxide–poly(3,4-ethylenedioxythiophene) composite through electrodeposition for non-enzyme glucose sensing. J. Mater. Chem. B.

[42] Wen Y.Y., Meng W., Li C., Dai L., He Z.X., Wang L., Li M., Zhu J. (2018). Enhanced glucose sensing based on a novel composite Co II -MOF/Acb modified electrode. Dalton Trans..

[43] Lin X.Y., Wang Y.F., He W.H., Ni Y.N., Kokot S. (2017). Nano-composite of Co_3_O_4_ and Cu with enhanced stability and catalytic performance for non-enzymatic electrochemical glucose sensors. RSC Adv..

[44] Ma L., Wang X.Y., Zhang Q.R., Tong X.L., Zhang Y., Li Z.A. (2018). Pt catalyzed formation of a Ni@Pt/reduced graphene oxide nanocomposite: preparation and electrochemical sensing application for glucose detection. Anal. Methods.

[45] Kano K., Takagi K., Inoue K., Ikeda T., Ueda T. (1996). Copper electrodes for stable subpicomole detection of carbohydrates in high-performance liquid chromatography. J. Chromatogr. A.

[46] Kano K., Torimura M., Esaka Y., Goto M., Ueda T. (1994). Electrocatalytic oxidation of carbohydrates at copper(ii)-modified electrodes and its application to flow-through detection. J. Electroanal. Chem..

[47] Gou X.F., Sun S.D., Yang Q., Li P.J., Liang S.H., Zhang X.J., Yang Z.M. (2018). A very facile strategy for the synthesis of ultrathin CuO nanorods towards non-enzymatic glucose sensing. New J. Chem..

[48] Sahoo R.K., Das A., Samantaray K., Singh S.K., Mane R.S., Shin H.C., Yun J.M., Kim K.H. (2019). Electrochemical glucose sensing characteristics of two-dimensional faceted and non-faceted CuO Nanoribbons. Cryst. Eng. Comm..

[49] Yang A.L., Wang Y.J., Li S.P., Bao X.C., Yang R.Q. (2014). Stepwise synthesis of cuprous oxide nanoparticles with adjustable structures and growth model. Sci. China Technol. Sci..

[50] Cao H.M., Yang A.L., Li H., Wang L.L., Li S.P., Kong J.L., Bao X.C., Yang R.Q. (2015). A non-enzymatic glucose sensing based on hollow cuprous oxide nanospheres in a Nafion matrix. Sens. Actuators B.

[51] Akhavan A., Kalhor H.R., Kassaee M.Z., Sheikh N., Hassanlou M. (2010). Radiation synthesis and characterization of protein stabilized gold nanoparticles. Chem. Eng. J..

[52] Tsai D.H., DelRio F.W., Keene A.M., Tyner K.M., MacCuspie R.I., Cho T.J., Zachariah M.R., Hackley V.A. (2011). Adsorption and conformation of serum albumin protein on gold nanoparticles investigated using dimensional measurements and in situ spectroscopic methods. Langmuir.

[53] Das K., Kundu S. (2015). Adsorption and conformation variation of BSA protein with the size variation of the metallic nanoparticles in LB film. Coll. Surf. A Physicochem. Eng. Asp..

[54] Chakraborty S., Joshi P., Shanker V., Ansari Z.A., Singh S.P., Chakrabarti P. (2011). Contrasting effect of gold nanoparticles and nanorods with different surface modifications on the structure and activity of bovine serum albumin. Langmuir.

[55] Shi X.J., Li D., Xie J., Wang S., Wu Z.Q., Chen H. (2012). Spectroscopic investigation of the interactions between gold nanoparticles and bovine serum albumin. Chin. Sci. Bull..

[56] Mathew T.V., Kuriakose S. (2013). Studies on the antimicrobial properties of colloidal silver nanoparticles stabilized by bovine serum albumin. Coll. Surf. B Biointerf..

[57] Wu X., He X.X., Wang K.M., Xie C., Zhou B., Qing Z.H. (2010). Ultrasmall near-infrared gold nanoclusters for tumor fluorescence imaging in vivo. Nanoscale.

[58] Hu C.Y., Yang D.P., Wang Z.Y., Yu L.L., Zhang J.L., Jia N.Q. (2013). Improved EIS performance of an electrochemical cytosensor using three-dimensional architecture Au@BSA as sensing layer. Anal. Chem..

[59] Hu C.Y., Yang D.P., Wang Z.H., Huang P., Wang X.S., Chen D., Cui D.X., Yang M., Jia N.Q. (2013). Bio-mimetically synthesized Ag@BSA microspheres as a novel electrochemical biosensing interface for sensitive detection of tumor cells. Biosens. Bioelectron..

[60] Habeeb Muhammed M.A., Verma P.K., Pal S.K., Retnakumari A., Koyakutty M., Nair. S., Pradeep T. (2010). Luminescent quantum clusters of gold in bulk by albumin-induced core etching of nanoparticles: Metal ion sensing, metal-enhanced luminescence, and biolabeling. Chemistry.

[61] Hu C.Y., Yang D.P., Zhu F.J., Jiang F.J., Shen S.Y., Zhang J.L. (2014). Enzyme-labeled Pt@BSA nanocomposite as a facile electrochemical biosensing interface for sensitive glucose determination. ACS Appl. Mater. Interf..

[62] Xiao J.R., Li Y.W., Jiang A.H. (2011). Structure, optical property and thermal stability of copper nitride films prepared by reactive radio frequency magnetron sputtering. J. Mater. Sci. Technol..

[63] Zhang X.J., Wang G.F., Zhang W., Wei Y., Fang B. (2009). Fixture-reduce method for the synthesis of Cu_2_O/MWCNTs nanocomposites and its application as enzyme-free glucose sensor. Biosens. Bioelectron..

[64] Marioli J.M., Kuwana T. (1992). Electrochemical characterization of carbohydrate oxidation at copper electrodes. Electrochim. Acta.

[65] Retnakumari A., Setua S., Menon D., Ravindran P., Muhammed H., Pradeep T., Nair S., Koyakutty M. (2010). Molecular-receptor-specific, non-toxic, near-infrared-emitting Au cluster-protein nanoconjugates for targeted cancer imaging. Nanotechnology.

[66] Wang F., Yang Z.L., Zhou Y., Weng S.F., Zhang L., Wu J.G. (2006). Influence of metal ions on phosphatidylcholine–bovine serum albumin model membrane, an FTIR study. J. Mol. Struct..

[67] Lu N., Shao C.L., Li X.H., Shen T., Zhang M.Y., Miao F.J., Zhang P., Zhang X., Wang K.X., Zhang Y. (2014). CuO/Cu_2_O nanofibers as electrode materials for non-enzymatic glucose sensors with improved sensitivity. RSC Adv..

[68] Foo K.Y., Hameed B.H. (2010). Insights into the modeling of adsorption isotherm systems. Chem. Eng. J..

[69] Wang M.J., Song B., Liu J.L., Hua C.G., Wei D.P., Wong C.P. (2017). Precisely quantified catalyst based on in situ growth of Cu_2_O nanoparticles on a graphene 3D network for highly sensitive glucose sensor. Sens. Actuators B Chem..

[70] Yazid S.N.A.M., Isa I.M., Norhayati N. (2016). Novel alkaline-reduced cuprous oxide/graphene nanocomposites for non-enzymatic amperometric glucose sensor application. Mat. Sci. Eng. C Mater..

[71] Khedekar V.V., Bhanage B.M. (2016). Simple electrochemical synthesis of cuprous oxide nanoparticles and their application as a non-enzymatic glucose sensor. J. Electrochem. Soc..

[72] Chen A., Ding Y., Yang Z.M., Yang S.C. (2015). Constructing heterostructure on highly roughened caterpillar-like gold nanotubes with cuprous oxide grains for ultrasensitive and stable nonenzymatic glucose sensor. Biosens. Bioelectron..

[73] Luo Z.J., Han T.T., Qu L.L., Wu X.Y. (2012). A ultrasensitive nonenzymatic glucose sensor based on Cu_2_O polyhedrons modified Cu electrode. Chin. Chem. Lett..

[74] El Khatib K.M., Abdel Hameed R.M. (2011). Development of Cu_2_O/Carbon Vulcan XC-72 as non-enzymatic sensor for glucose determination. Biosens. Bioelectron..

